# ICSI Outcome in Infertile Couples with Different Causes of Infertility: A Cross-Sectional Study

**Published:** 2013-07-31

**Authors:** Mahnaz Ashrafi, Shahideh Jahanian Sadatmahalleh, Mohammad Reza Akhoond, Firouzeh Ghaffari, Zahra Zolfaghari

**Affiliations:** 1 Department of Endocrinology and Female Infertility at Reproductive Biomedicine Research Center, Royan Institute for Reproductive Biomedicine, ACECR, Tehran, Iran; 2 Department of Obstetrics and Gynecology, Faculty of Medicine, Tehran University of Medical Science, Tehran, Iran; 3 Department of Midwifery and Reproductive Health, Faculty of Medical Sciences, Tarbiat Modares University, Tehran, Iran; 4 Department of Epidemiology and Reproductive Health at Reproductive Epidemiology Research Center, Royan Institute for Reproductive Biomedicine, ACECR, Tehran, Iran

**Keywords:** ICSI, Pregnancy Rate, infertility

## Abstract

**Background:**

Different success rate of Intracytoplasmic Sperm injection (ICSI) has been observed in various causes of infertility. In this study, we evaluated the relation between ICSI outcome and different causes of infertility. We also aimed to examine parameters that might predict the pregnancy success rate following ICSI.

**Materials and Methods:**

This cross sectional study included1492 infertile women referred to Infertility Center of Royan Institute between 2010 and 2011. We assigned two groups including pregnant (n=504) and non-pregnant (n=988), while all participants underwent ICSI cycles. All statistics were performed by SPSS program. Statistical Analysis was carried out using Chi-square and t test. Logistic regression was done to build a prediction model in ICSI cycles.

**Results:**

The overall clinical pregnancy rate in our study was 33.9% (n=1492). There was a statistically significant difference in mean serum concentration on day 3 after application of luteinizing hormone (LH) between the pregnant and the non-pregnant groups (p<0.05). However, There were no significant differences between two groups in the serum concentrations on day 3 after application of the following hormones: folliclestimulating hormone (FSH), thyroid-stimulating hormone (TSH), and metoclopramidestimulated prolactin (PRL) . We found no association between different causes of infertility and clinical outcomes . The number of metaphase II (MII) oocytes, embryo transfer, number of good embryo (grade A, B, AB), total dose of gonadotropin, endometrial thickness, maternal age, number of previous cycle were statistically significant between two groups (p<0.05).

**Conclusion:**

Our results indicate that ICSI in an effective option in couples with different causes of infertility. These variables were integrated into a statistical model to allow the prediction for the chance of pregnancy following ICSI cycles. It is required that each infertility center gather enough information about the causes of infertility in order to provide more information and better assistance to patients. Therefore, we suggest that physicians prepare adequate training and required information regarding these procedures for infertile couples in order to improve their knowledge.

## Introduction

Intracytoplasmic Sperm injection (ICSI) brings an operative technology in the fields of assisted reproduction technology (ART) (1). Nowadays, this way becomes one of the most important and efficient treatment approaches which is used by infertility clinics. One of problems in infertility centers is low rate of pregnancy in ICSI cycles (2). There is still an ongoing debate among reproductive embryologists and endocrinologists about using ICSI for the treatment of the infertile couples. There is a general agreement that ICSI has become a gold standard technique for the treatment of male factor infertility (3, 4), but many physicians recommend ICSI or invitro fertilization (IVF) to patients with tubal factor infertility (5). However, there is controversy about the replacement of ICSI with IVF in women with sever tubal factor infertility (5, 6). It could be helpful in planning strategies for the treatment of infertile couples with unexplained infertility (7, 8). Although ICSI has been applied for the male factor infertility more than tubal factor infertility and endometriosis, it may be a useful technique to overcome these types of fertilization defect in women (9, 10).

Multiple studies have also showed a higher risk of congenital abnormalities, cardiovascular, musculoskeletal defect, low birth weight, preterm delivery and increase perinatal mortality in IVF/ ICSI offspring (11-15). Nevertheless, there is an imperfect image of the risk factors associated with the application of ICSI about offspring. Although this technique is accepted as a standard routine for couples with different causes of infertility (11). There are no data to suggest that ICSI is a proper method in order to apply for all ART cases. Since ICSI has a great cost to both couple and the health care system, it is necessary to assess its efficacy (3).

Different success rate of ICSI has also been observed in various causes of infertility. Our prediction model has been developed in reproductive medicine to help gynecologists in assessing the chances of pregnancy following ICSI. With these models, gynecologists can calculate the probability of a treatment pregnancy as well as the probability of pregnancy success with ICSI. In this study, we evaluated the relation between ICSI outcome and special cause of infertility. We also aimed to examine parameters that might predict pregnancy success rate following ICSI.

## Materials and Methods

This cross sectional study included 1492 infertile women referred to Infertility Center of Royan Institute between 2010 and 2011.We assigned two groups including pregnant (n=504) and nonpregnant (n=988), while all participants underwent ICSI cycles.

Information about age, menstrual duration, number of previous cycle, endometrial thickness, number of embryo transfer, embryo quality, duration of infertility, type of infertility, cause of infertility (ovulatory factor, unexplained factor, male factor...) were obtained from patient’s file, then the collected data were analyzed and compared between the two groups. Inclusion criteria were as follows: male infertility, ovarian infertility (including polycystic ovary syndrome (PCOS) and diminished ovarian reserve), tubal infertility, unexplained infertility, recurrent abortion, and endometriosis.

The couples with testicular atrophy, anatomical abnormalities, infection, uterine fibroids, systemic disease and history of ICSI/IVF failure more than three times were excluded from the study. In all participants, serum follicle-stimulating hormone (FSH; Pishtaz-Tab, Tehran, Iran) and luteinizing hormone (LH; Pishtaz- Tab, Tehran, Iran) were measured on day 3 of the cycle preceding ovarian stimulation. The ovarian stimulation protocol for all patients was performed according to the standard long protocol. Both groups started with Bucereline acetate (Superfact; Aventis Pharma Deutshlan, Frankfurt, Germany) 500 μg on day 21 of the previous cycle and continued daily until the day of hCG administration. After ovarian stimulation, injection of 10000 IU of human chorionic gonadotropin (hCG; Choriomon; IBSA, Switzerland) was given when at least two follicles ≥18 mm were detected. Transvaginal follicular aspiration was performed under ultrasound guidance, 34-36 hours after the administration of hCG. Afterwards, ICSI cycle was completed. All patients received luteal phase support through daily administration of 100 mg natural progesterone (sterop Laboratories, Brussls, Belgium) and progesterone up to 8 week of gestation. A clinical pregnancy was confirmed by the observation of gestational sac in ultrasonography. This study was approved by the Royan Ethics Committee.

In addition, all subjects agreed to participate in the study were required to sign a consent form approved by the Royan Ethics Committee.

In order to build a prediction model, we used backward logistic regression analysis, in which a p-value of 0.15 was used as an entry criterion, whereas a p-value of 0.10 was the threshold for a variable to stay in the model. The performance of the model was calculated as the area under the receiver operating characteristic (ROC) area under roc curve (AUC). An AUC of 0.5 indicates no discriminative performance, whereas an AUC of 1.0 indicates perfect discrimination.

Calibration of the model was assessed by comparing the predicted probability of pregnancy in a category of patients with the observed percentage of pregnant woman in the same category. We first categorized the predicted probabilities of pregnancy in 10 groups, and then we compared the mean predicted probability of pregnancy within a group with the observed probability of the same group.

### Statistical analysis

All statistical analysis was performed by SPSS program (Version 18; USA). Chi-square and t test were used for analysis. Also, In order to predict the result of ICSI, we used logistic regression. The data were expressed as means ± standard deviation (SD). Odds ratio (OR) and 95% confidence interval (95% CI) were also calculated for each factor. The value of p<0.05 was considered to be statistically significant.

## Results

In this study, 1492 women were enrolled. The mean maternal age was 32.3 ± 5.3 years, while the mean duration of infertility was 7.2 ± 5.07 years. Of 1492 women, 1172 (78.5%) individuals were with primary infertility, while 320 (21.5%) individuals were with secondary infertility. Overall, 59.8% of patients had previous treatment for infertility, and we also found a significant reduction of pregnancy rate in the group with previously failed ICSI attempts. The general characteristics of all participants undergoing ICSI, divided into pregnant and non-pregnant groups,are provided in table 1.

Our findings confirmed that the pregnancy rate reduced when the woman’s age increased (OR=0.93, 95% CI=0.91-0.95). In addition, our result revealed that pregnancy rate were lower in primary infertility than secondary infertility, but it is n’t significant. Our results also showed no significant effect of body mass index (BMI) on pregnancy rate (Table 1).

**Table 1:** Characteristics of women undergoing ICSI in two groups of pregnant and non-pregnant


		Pregnant	Non-pregnant	OR (CI 95%)*	Significant

Age (Y)**		31.1 ± 4.8	32.9 ± 5.4	0.93 (0.91-0.95)	<0.0001a
**Type of infertility**	Primary-N (%)	396 (33.8%)	776 (66.2%)	1***	
	Secondary-N (%)	109 (34%)	211 (66%)	1.005 (0.774-1.30)	0.969b
BMI (Kg/m^2^)*		25.8 ± 3.7	26.03 ± 3.7	0.98 (0.95-1.01)	0.37a
No. previous cycle*		0.46 ± 0.75	0.66 ± 0.99	0.76 (0.67-0.87)	<0.0001a
**Menstrual duration**		6.25 ± 1.5	6 ± 1.3	1.12 (1.04-1.21)	0.002a


*; OR=Odds ratio, CI=Confidence interval, **; Values are mean ± SD, ***; Reference category, a; Independent sample t test and b; Chi-square test.

Table 2 indicates the different causes of infertility and their likelihood of occurrence, like ovulatory factor (7.4%), tuboperitoneal factor (5.2%), unexplained factor (10%), male factor (59.1%), recurrent abortion (2.1%), uterine factor (0.4%), Mix (14%), and others (impotency, vaginismus, genetic disorder, etc) (1.7%). Furthermore, table 2 shows outcome of ICSI cycles in different causes of infertility, where as there was no statistically significant difference between groups.

Cycle characteristics are depicted in table 3. We found total dose of gonadotropin of nonpregnant group to be significantly higher than that of pregnant group (p<0.0001). Table 3 reveals that there was statistically significant difference between the pregnant and non-pregnant groups in endometrial thickness (p<0.05). The number of retrieved metaphase II (MII) oocytes was significantly higher in pregnant group than that in non-pregnant group. There was no significant difference between two groups in the mean serum concentrations on day 3 after application of the following hormones: FSH (7.04 ± 3.43 IU/ml), LH (5.51 ± 4.06 IU/ml), TSH (2.35 ± 1.81 IU/ml), metoclopramide-stimulated prolactin (PRL) (163.40 ± 264.82 IU/ml) (Table 3).

**Table 2:** Success rate of ICSI outcome in infertile couples with different cause of infertility


	Pregnant % (N)	Non-pregnant % (N)	P value a

**Ovulatory factor**	29.7 (33)	70.3 (79)	0.936
**Tuboperitoneal factor**	32.2 (25)	67.8 (53)	
**Unexplained factor **	34 (50)	66 (99)	
**Male factor**	35 (307)	65 (573)	
**Recurrent abortion**	31.2 (10)	68.8 (22)	
**Uterine factor **	50 (4)	50 (4)	
**Mix**	32.9 (68)	67.1 (141)	
Other*	37.5 (9)	62.5 (15)	


*; Impotency, vaginismus, genetic disorder and a; Chi-square test.

**Table 3:** Table 3: Cycle parameters of the patients undergo ICSI


Pregnant Non-pregnant	OR* (CI 95%)	Significant a

Total dose of gonadotropin **	1986.48 ± 941	2240.96 ± 1035	0.9997 (0.9996 ± 0.9998)	<0.0001
Endometrial thickness **	9.87 ± 1.7	9.57 ± 1.8	1.09 (1.03 - 1.16)	0.002
No. of MI oocytes **	0.4 ± 0.8	0.4 ± 0.8	0.97 (0.85 - 1.11)	0.68
No. of MII oocytes **	8.3 ± 3.9	7.2 ± 4.1	1.06 (1.04 - 1.09)	<0.0001
Fertilization rate **	0.71 ± 0.2	0.68 ± 0.3	1.31 (0.92 - 1.89)	0.13
No. of good embryo (A, B, AB) **	2.1 ± 2.9	1.5 ± 2.4	1.08 (1.03 - 1.12)	<0.0001
No. of embryo transfer **	2.50 ± 0.66	2.36 ± 0.79	1.29 (1.11 ± 1.48)	<0.0001
Serum FSH level on day 3 (IU/ml) **	6.81 ± 3.5	7.13 ± 3.4	0.97 (0.94 - 1.005)	0.10
Serum LH level on day 3 (IU/ml) **	5.82 ± 4.81	5.35 ± 3.8	1.02 (1 - 1.05)	0.04
Serum TSH level on day 3 (IU/ml) **	2.35 ± 1.71	2.37 ± 1.8	0.99 (0.93 - 1.05)	0.85
Serum PRL level on day 3 (IU/ml) **	163.42 ± 232.1	162.88 ± 270.3	1.00 (1.00 - 1.00)	0.97


* ; OR=Odds ratio, CI=Confidence interval, **; Values are mean ± SD and a; Chi-square test.

However, there was statistically significant difference in the mean serum level on day 3 after application of LH between the pregnant and the non-pregnant groups. There were no statistically significant differences between groups in number of MII oocytes, embryo transfer and number of good embryo (grade A, B, AB) (Table 3). No significant difference was also observed between the pregnant and non-pregnant groups in the number of metaphase I (MI) (OR= 0.97, CI=0.85-1.1; p=0.68). Fertilization rate in the pregnant and non-pregnant groups was 71 and 68%, respectively (OR=1.31, CI=0.92- 1.89; p=0.13) (Table 3).

Finally, in order to build a prediction model and find the most important factors that affect pregnancy rate, we used a logistics regression model in a backward manner. Table 4 shows the result of fitting logistic regression model to the data.

Age, menstrual duration, number of previous cycle, endometrial thickness, number of embryo transfer, and embryo quality in the logistic regression model were significantly associated with pregnancy outcome. Age and number of previous cycle were negatively associated with pregnancy outcome, while menstrual duration, endometrial thickness, embryo quality and number of embryos transferred were positively associated with pregnancy outcome (Table 4).

**Table 4:** Table 4: Result of logistic regression analysis


Variable	OR*	95% CI	Significant

**Age (Y)**	0.942	0.920-965	<0.0001
**Menstrual duration (Day)**	1.110	1.023-1.206	0.013
**The number of previous cycle**	0.838	0.721-.973	0.021
**Endometrial thickness**	1.090	1.021-1.163	0.010
**No. of embryo transfer **	1.092	1.051-1.135	<0.0001
**No. of good embryo transfer (A, B, AB)**	1.448	1.280-1.639	<0.0001
**Constant**	0.271		0.025
	AUC: 0.681 (95% CI 0.653-0.709)		


* OR; Odds ratio and CI; Confidence interval.

**Fig 1:**
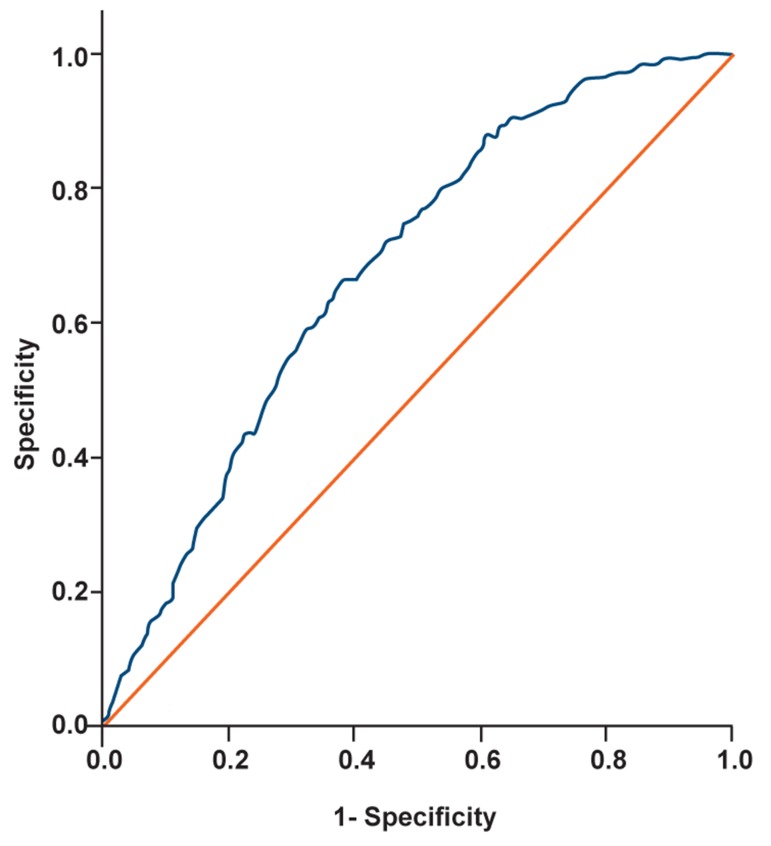
ROC curve for assessment discrimimative preformance of logistic regression.

AUC shows the discriminative performance of the logistic model. The AUC of 0.5 shows no discriminative performance, while AUC of 1.0 indicates perfect discrimination. The AUC for the fitted logistic model was 0.681 (95% CI=0.653-0.709) that shows good predictive performance (Fig 1).

**Fig 2:**
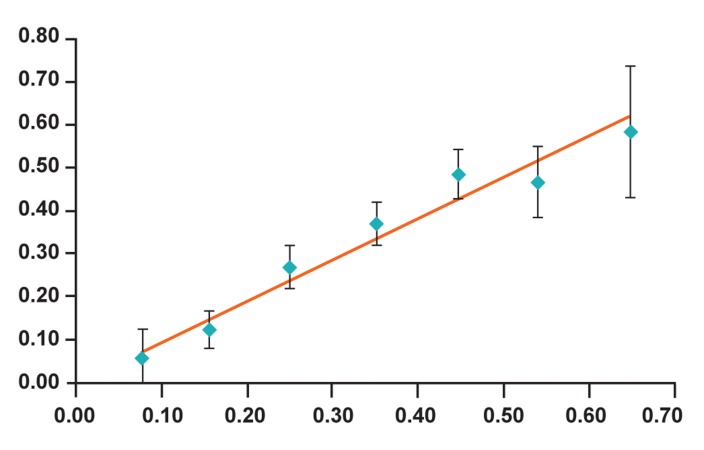
Calibration plot, showing the relationship between predicted and observed rate of pregnancy after ICSI.

Figure 2 indicates the calibration of the prediction model for pregnancy after ICSI. The predictive performance of the model is acceptable because the 95% confidence intervals of the observed pregnancy rates overlap with the predicted pregnancy rates.

## Discussion

Although ICSI is originally developed to treat male infertility, it has been used for infertile couples with different causes of infertility (1). We evaluated the relation between ICSI outcome and different causes of infertility, while our obtained data illustrated the different success rate of ICSI in various causes of infertility (Table 2). Our data also supported maternal age as an important predictor for having a successful outcome in ICSI, which is found to bein agreement with results of some studies (16-20), while contradicts to a study of Spandorfer et al. in which they have found no impact of paternal age on ICSI success rates (21). In the present study, endometrial thickness was regarded as prognostic parameters for successful pregnancy in ICSI, which was in agreement with several studies showing endometrial thickness was greater in cycles resulting in pregnancy than in cycles not resulting in pregnancy (16, 19, 20, 22, 23). In addition, other studies have showed increased endometrial thickness is not associated with decreased pregnancy rates in ART treatment (24, 25).

In our study, no significant difference was found in BMI between pregnant and not-pregnant groups. Usoniene et al. reported the same results of pregnancy rate for different BMI groups (19). The other studies indicated that when BMI was higher than 25 kg/m^2^, the pregnancy rate was significantly lower (26, 27). Non-PCOS patients undergoing IVF/ICSI showed an increase in BMI independently of age, LH, FSH, as well as duration and type of infertility, whereas these factors affect pregnancy rate, significantly (28).

In our study, mean LH serum concentration in pregnant group were significantly higher than that in non-pregnant group (OR=1.02, CI=1-1.05; p=0.04). At the beginning of stimulation, high LH concentration can lead to increased endometrial maturation at oocyte pick-up (29). Balasch et al. found LH is not necessary for follicular growth, but externally administered LH possibly shows a primary role in complete maturity of the oocyte and follicle (30). LH is required for normal folliculogenesis, while low LH concentrations on day 3 could lead to a poor ovarian response (31). However, basal LH and E2 levels are not considered as proper factors in order to distinguish the infertile patients responding differently to ovarian stimulation (32).

History of menstrual cycle length (MCL) will be used as a simple sign of ovarian reserve, which is primarily determined by the growth rates and quality of ovarian follicles. Our result showed women with menstrual duration more than 6 days had more chance of pregnancy than those with cycles less than 6 days (OR=1.12, CI=1.04-1.21; p=0.002). Shortening of MCL causes an abbreviated follicular phase, but in general, luteal phase length is preserved (33). The shorter follicular phase is associated with a decrease in inhibin B and an increase in FSH, which could be due to smaller number of antral follicles (33).

Tomas et al. found the influence of embryo quality in pregnancy prediction (34). Our result showed higher pregnancy rate in the best quality of embryo transferred. Different reports have also revealed that ICSI may be performed successfully incases with a history of fertilization failure (35). We also found that women with repeated fertilization failure were at higher risk of pregnancy loss (Tables 2, 4).

Esinler et al. showed that larger doses of gonadotropin are required for overweight and obese women (36). Our findings also revealed the significant effects of total dose of gonadotropin and endometrial thickness on outcome of pregnancies conceived by ICSI.

It is evident that various factors may influence the outcome of ICSI. Kovacs et al. showed, women who became pregnant after ART showed thicker endometrium, better quality of embryo, as well as more follicles, oocytes and embryos (25) as we observed in our study. In this study, we examined variable parameters in patients with different causes of infertility and predicted pregnancy success rates following ICSI. In addition, patient characteristics, total dose of gonadotropin, endometrial thickness, number of previous cycle and quality of embryo transferred were evaluated as predictors of success rates following ICSI. Our findings confirmed that larger doses of gonadotropin, a decrease in endometrial thickness and in appropriate embryo quality reduced significantly pregnancy success rate. Our results also indicated that ICSI in an effective option in couples with different causes of infertility. We did not find an association between cause of infertility and clinical outcomes. The overall pregnancy rate in our study was 33.9% (n=1492).

Undoubtedly, this database was not large enough to allow definite conclusion, and needed further supports to continue the follow-up of pregnancy outcome after ICSI.

Our models can be used reliably as a guide for making decisions for fertility management in infertile patients. The effects of using these models in patient care need further experimental investigation.

## Conclusion

ICSI is an important treatment option for various indications of infertility. The present study showed that pregnancy rate affected by the number of previous cycle, total dose gonadotropin, endometrial thickness, number of previous cycle, quality of embryo transferred and menstrual duration. It is required that each infertility center gather enough information about the causes of infertility in order to provide more information and better assistance to patients. Therefore, we suggest that physician sprepare adequate training and required information regarding these procedures for infertile couples in order to improve their knowledge.
